# Who and How Many? Using Integrated Health and Education Data to Study Early Care Experiences of Children in the US

**DOI:** 10.23889/ijpds.v11i2.3366

**Published:** 2026-08-17

**Authors:** Quentin H. Riser, Heather L. Rouse, Hannah Kim, Cassandra J. Dorius, Jessica Bruning, Ji Young Choi, William T. Abraham

**Affiliations:** 1 Institute for Research on Poverty, University of Wisconsin-Madison; 2 School of Human Ecology, University of Wisconsin-Madison; 3 Human Development and Family Studies, Iowa State University; 4 Crane Center for Early Childhood Research, The Ohio State University; 5 Human Development and Family Science, The Ohio State University

**Keywords:** Integrated Data Systems, Early Care and Education, School Readiness, Social Determinants of Health, Educational Equity

## Abstract

Fragmentation across early care and education (ECE) systems in the United States obscures how many children access services and how these experiences shape school readiness. This study leverages Iowa’s Integrated Data System for Decision-Making (I2D2), which links health, social determinants of health (SDOH), and education records, to provide one of the first unduplicated counts of ECE participation statewide. Drawing on a matched cohort of 27,321 children eligible for kindergarten in 2017–2018, we examine three aims: (1) describe patterns of centre-based ECE participation; (2) assess associations between child/family characteristics and participation; and (3) evaluate links between ECE and kindergarten suspensions and attendance. Results indicate that 73% of children participated in at least one ECE program prior to school entry, with substantial overlap across public and private preschool, and subsidised or unsubsidised child care. ECE participation varied as a function of poverty, race/ethnicity, maternal education, and cumulative early-life risks. ECE participation was associated with better kindergarten attendance but not suspension. Findings underscore both the promise of integrated data for advancing equity-driven research and the need for policies that target families with the greatest barriers to ECE access.

## Introduction

Integrated data systems (IDS) have emerged as an important innovation for addressing fragmentation across public service sectors and improving the capacity to guide policy and practice. By linking individual-level administrative records across health, social services, housing, and education, IDS make it possible to examine population trends, identify risk and protective factors, and better understand service engagement across systems [1, 2]. These capacities are particularly relevant in early childhood, a period when experiences within families, communities, and early learning environments jointly shape development. Yet public schools often receive children with limited information regarding their early educational participation or broader social determinants of health (SDOH), such as economic stability or housing conditions, that influence school readiness [3, 4]. Linking early care and education (ECE), health, and SDOH data offers a more complete picture of children’s developmental contexts and supports efforts to design instructional and support strategies that are responsive to children’s needs [5, 6].

Despite the potential of IDS, the ECE sector in the United States remains highly fragmented, with programs operating across multiple agencies, funding streams, and governance structures that rarely align with one another or with the K–12 systems that later serve these children [7, 8]. This fragmentation makes it difficult to determine which children access which services, how experiences accumulate across settings, and how early opportunities relate to later outcomes [9].Prior studies have contributed important insights through retrospective surveys, single-program evaluations, and national longitudinal datasets such as the Early Childhood Longitudinal Study- Birth Cohort (ECLS-B) and National Survey of Early Care and Education (NSECE); however, these approaches often capture only partial segments of children’s experiences and may rely on dated data, limiting their ability to fully represent entire state populations [10–12].

Although international studies demonstrate how IDS can inform policy in other sectors [13, 14], applications focused on children’s pathways into formal schooling remain limited. In the US, administrative data have also been leveraged in adjacent policy domains, including research on child support payments [15], children and families experiencing homelessness [16], and child welfare system involvement [17]. This work illustrates how administrative data can generate timely, population-level insight that supports program learning and improvement.

The present study continues this kind of work by examining a statewide IDS that links health, social services, and ECE data, demonstrating how such a system can be used to generate actionable, population-level evidence to inform early education policy and practice [18–21]. Guided by ecological and cumulative disadvantage frameworks, this study examines how early developmental contexts shape children’s ECE participation and kindergarten adjustment. Ecological theory emphasises that children’s early competencies develop through proximal processes embedded within layered environments, such that family resources, caregiver wellbeing, and early health conditions can support–or constrain—development [22–24]. Cumulative disadvantage theory further posits that early risks tend to cluster and compound over time, generating constraints potentially beyond any single exposure and contributing to widening school readiness gaps as children encounter the demands of formal schooling [25–27]. Consistent with this perspective, we leverage linked administrative data to operationalise early-life health and SDOH risks as both individual indicators and a cumulative risk index (0–5), enabling cohort-level measurement of multidimensional disadvantage that is rarely possible outside integrated data systems.

In the present study, predictors were derived from public health vital statistics at birth and capture socioeconomic and maternal resource disadvantage (poverty proxy via Medicaid/ The Special Supplemental Nutrition Program for Women, Infants, and Children (WIC); unmarried mother; low maternal education; teen mother) and early health/access risks (preterm/low birthweight; inadequate prenatal care; prenatal smoking) [28–30]. In addition to examining these indicators individually, we constructed a cumulative risk index (0–5) summarising accumulation of disadvantage across domains, consistent with cumulative risk research documenting incremental associations between risk exposure and early academic and behavioural outcomes [31, 32].

For outcomes, the present study includes an unduplicated indicator of any centre-based early care and education participation in the year prior to kindergarten entry. In Iowa, children access centre-based early learning through several pathways, including subsidised child care, state-supported preschool offered through school districts, federally funded Head Start, and community-based private child care centres.

To capture these pathways, early care and education participation was constructed by integrating three statewide administrative data sources: child care subsidy payment records, which identify children receiving state or federal subsidy support for centre-based care; Department of Education preschool enrollment records, which capture participation in publicly funded early childhood programs offered through school districts, including state preschool, Shared Visions, and IDEA preschool services; and Teaching Strategies GOLD records, which capture enrollment in programs participating in Iowa’s statewide preschool assessment system, including district-affiliated prekindergarten, Head Start grantees, and many community-based providers.

Because each data source captures a somewhat different segment of Iowa’s early care landscape, we conceptualise children’s participation in broader care constellations rather than relying on program acronyms alone. Children appearing across all three systems can be understood as participating across multiple early care systems, likely reflecting families who combine publicly funded preschool, child assessment-linked programs, and child care subsidies to construct full-day care arrangements. Children appearing in public preschool and assessment records, but not subsidy records, may represent public prekindergarten participants who do not rely on child care subsidies. Children appearing only in the assessment system may include those in private or community-based early learning settings outside the state’s voluntary preschool and subsidy systems. Finally, children appearing only in subsidy records represent a small but important group of subsidy-supported children who are not captured in public preschool or statewide assessment records.

We anticipated that associations with child and family characteristics would vary across these care constellations in both direction and magnitude. For instance, subsidy-supported participation may be more closely associated with socioeconomic disadvantage and early-life risk exposure, given that eligibility is income-based and participation reflects subsidised care use. In contrast, participation in public preschool without subsidy reliance may show weaker or different associations with disadvantage, reflecting broader access through district-based preschool programs and variation in local availability. Assessment-only participation may capture a mixed group of children in community-based, private, or Head Start-affiliated settings, meaning that observed associations could fall between those for public preschool and subsidy-supported care or vary depending on which programs contribute records. Taken together, this integrated approach allows us to move beyond program silos and identify meaningful patterns of early care participation across education, assessment, and subsidy systems.

Research using publicly monitored risk indicators shows that early-life risks are common, co-occur, and are incrementally and negatively related to school outcomes when operationalised cumulatively [31, 32]. Moreover, ECE participation is not randomly distributed: subsidy receipt and enrollment in publicly supported ECE programs are related to socioeconomic and family risk, underscoring the importance of examining how early-life SDOH and health indicators relate to ECE participation patterns [33]. Prior evidence also suggests that centre-based preschool participation is associated with improved school engagement during the transition to kindergarten; for example, children attending centre-based prekindergarten have lower odds of chronic absenteeism in kindergarten [34], and children enrolled in Head Start or public prek show lower kindergarten absence rates than non-participants [35]. Collectively, this literature supports examining both individual and cumulative early-life risks as predictors of ECE participation and testing whether ECE participation is associated with kindergarten attendance and disciplinary outcomes after accounting for early-life disadvantage.

As such, the study was guided by three aims. Aim 1 uses linked administrative records to document ECE participation patterns for an entire cohort of kindergarten entrants, including unduplicated counts across ECE systems. Aim 2 assesses how early-life health and SDOH characteristics–both individual risks and cumulative risk–relate to children’s ECE experiences. Aim 3 evaluates whether ECE participation is associated with kindergarten suspensions and attendance while accounting for early-life health and SDOH risk. The first aim is exploratory in nature, as it seeks to characterise patterns of ECE participation across multiple systems and describe how children’s demographic and family risk profiles vary across those groups. Aligned with Aims 2 and 3, we have two hypotheses. For Aim 2, we hypothesise that children exposed to greater early-life disadvantage–particularly higher cumulative risk–will be less likely to participate in centre-based early care and education settings that require stable access, enrollment navigation, and schedule alignment, and more likely to appear in subsidy-supported or fragmented care participation patterns [31, 32, 36]. For Aim 2, we hypothesise that participation in an early care and education experience will be uniquely positively associated with lower odds of poor kindergarten outcomes, including poor attendance and exclusionary discipline, after controlling for early-life disadvantage, cumulative risk, and other child and family characteristics [37–39].

## Method

### Data System

The present study uses integrated administrative data from Iowa’s Integrated Data System for Decision-Making (I2D2) [18]. Following approved legal agreements and governance protocols, administrative records are collected annually from data contributors, cleaned, and stored in a secure server. Project proposals are reviewed by a governance board comprised of appointees from each system authorised to vet and consider projects as they align with agency priorities. Once projects are approved, individual files are integrated using a comprehensive set of deterministic and probabilistic matching algorithms using identifiers contained within the administrative data (e.g., first and last name, birth date) in a staged process. For this study, birth records were first matched to kindergarten enrollment records to generate a sample of children born in the state who also attended public school. Additional records from experiences that occurred anytime between birth and school entry were then integrated (e.g., subsidised child care, preschool). Prior to matching, rigorous cleaning and verification procedures were implemented, including checks for internal consistency and missing data. All personal identifiers were removed after the data integration was complete and the anonymised dataset was used for all analyses.

### Sample

The analytic sample was drawn from the full cohort of 39,200 children born in Iowa who were age-eligible to enter kindergarten in the 2017–2018 academic year. Of these, 27,321 children (69%) were successfully linked to public kindergarten enrollment records. The remaining 31% were not matched and likely include children who moved out of state prior to school entry, enrolled in private or homeschool settings not captured in state administrative data, or could not be linked due to inconsistencies in identifying information.

Among the 34,813 children enrolled in kindergarten statewide, the 27,321 matched cases represent 78% of the kindergarten population captured [40].

### Measures

To assess children’s early experiences and later outcomes, the study relied on a combination of individual-level indicators and cumulative risk indices derived from health systems (vital statistics birth records) and education systems (preschool assessment data).

#### Early Care and Education Participation

Three data sources provided information about ECE that were then combined to create an unduplicated indicator of whether or not children had any experience prior to kindergarten entry. Child Care Assistance (CCA) enrollment was collected from the Department of Health and Human Services who administers the state’s federal child care subsidy program. Children were classified as having participated in CCA if they were enrolled in the program and received at least one child care assistance payment for a centre-based care experience during the year prior to kindergarten entry. State-funded preschool (PreK) participation was indicated for any child found in the Department of Education enrollment records. Enrollment records included information about the child’s assigned school and the type of classroom attended, enabling identification of children who experienced a DE-funded early education setting prior to kindergarten entry. Other preschool experience was identified using a preschool assessment data system (Teaching Strategies GOLD). The state Department of Education funds a statewide licence for this system to allow any preschool program (e.g., public or private) access to use it for the children in their programs. It is required to be used by all state-funded programs and Head Start, and other programs also use it to track developmental outcomes. For this study, any child who received a GOLD assessment during the 2017-2018 school year was considered to have had a preschool experience. No other data from this system was used. Any ECE participation was then coded for any child with a record of involvement in at least one of the above during the year before kindergarten. This composite measure captures participation in a range of centre-based early learning settings across public and private programs.

#### Kindergarten Suspensions and Attendance

Two indicators were derived from records provided by the state Department of Education. Poor attendance was defined as attending less than 90% of school days in the kindergarten year. This was calculated as the proportion of days attended divided by days enrolled in kindergarten. Children falling below the 90% threshold were coded as having poor attendance (1) and 90% or above as not poor attendance (0), and reflects a cutoff frequently used in policy and research to identify problematic attendance that relates to continued disengagement and later achievement problems (US Department of Education, 2025). Suspension was indicated for any child who was removed from school for disciplinary reasons of any type, including inappropriate use of language, bullying, or dress code infractions. A dichotomous variable indicating suspension (1) or no suspension (0) during the kindergarten year was created.

#### SDOH Risks at Birth

Several variables collected from the public health vital statistics records were used to describe SDOH characteristics of children. These were used as both individual predictors and summed to create a cumulative SDOH risk indicator. Individual indicators were constructed from the birth records using established definitions from prior research. Poverty was a proxy variable comprising of indicators documenting receipt of Medicaid or WIC at birth–these are federal programs for low-income families with eligibility thresholds at 133% and 185% of the federal poverty line, respectively [30]. Unmarried mother was indicated for any child whose mother was not married, divorced, or widowed at the time of birth. Low maternal education was indicated for children whose mother was older than 19 but had completed less than 12 years of schooling. Teen mother was coded for children born to a mother under the age of 20. Preterm or low birth weight status was indicated for children born at less than 37 weeks gestation or with a birth weight under 2,500 grams [28]. Inadequate prenatal care includes children whose mother had fewer than four prenatal visits overall or no prenatal visits in the first trimester [29]. Prenatal smoking was indicated for children whose mother indicated tobacco use during pregnancy or in the three months prior to pregnancy. Cumulative risk was calculated by summing the presence of the seven individual SDOH risk variables for each child. Analysing the distribution of this variable showed a severe left skew and very few instances of risks above 4, so this variable was truncated into groups with 0, 1, 2, and 3 or more risks for analyses.

**Child and family characteristics** were included as covariates. Child sex was obtained from Vital Statistics birth records. Race/ethnicity were obtained from the Iowa Department of Education. Rurality was coded based on the county of residence at birth and classified according to prior literature [41]. Using this definition, Iowa has 88 rural counties (out of 99) identified as geographic areas comprised of census block groups having a population density of less than 1,000 people per square mile. Child age in months at the start of kindergarten was calculated by subtracting the birth date from the kindergarten age cutoff date (September 15^th^, 2017).

### Data Analysis

All analyses were conducted using Stata 16.0. We first computed descriptive statistics for demographic characteristics and for individual and cumulative SDOH risk indicators. As an initial diagnostic step, tolerance and variance inflation factor (VIF) values were examined, with results indicating no meaningful multicollinearity among predictors.

Analyses were guided by three research aims. To address Aim 1, which describes patterns of ECE participation and estimates the unduplicated number of children with at least one centre-based experience, we conducted a series of tabulations for each combination of ECE experiences as well as for the unduplicated count of children with any ECE participation. For Aims 2 and 3, we employed logistic regression models given the binary nature of the outcomes. Logistic regression models estimate the log-odds of an event as a function of observed predictors and are well suited for modelling dichotomous outcomes without assuming normality or homoscedasticity of errors [42]. For Aim 2, we estimated eight binary logistic regression models to examine the unique association of child and family characteristics with ECE participation. Four models predicted participation in each ECE category using individual birth risk indicators, and four parallel models used cumulative birth risk. In each model, the focal ECE category was compared with all children not in that category. This approach allowed us to estimate the relative contribution of each predictor–net of the other covariates–to the likelihood of participating in a given ECE group versus all other groups.

For Aim 3, we estimated four binary logistic regression models to examine whether ECE participation was associated with kindergarten outcomes. Two models predicted poor attendance and suspension using individual birth risk indicators, and two parallel models predicted the same outcomes using cumulative birth risk. In these models, ECE participation was the focal predictor, and models adjusted for child age, sex, race/ethnicity, parent immigration status, number of siblings, rurality, and early-life risk. This approach allowed us to examine whether any public preschool participation was uniquely associated with lower or higher odds of poor attendance and suspension after accounting for demographic characteristics, family context, rurality, and early-life disadvantage.

Across all models, results are presented as odds ratios. For Aim 2, odds ratios greater than 1.0 indicate higher odds of being in the focal ECE group, while values less than 1.0 indicate lower odds. For Aim 3, odds ratios greater than 1.0 indicate higher odds of poor attendance or suspension in kindergarten, while values less than 1.0 indicate lower odds of those outcomes.

## Results

As shown in Table 1, the sample is fairly representative of children born in the state, as well as the state’s public school population. Among matched children, 51% were male, and the majority were White (78%), followed by Hispanic (10%), Black (5%), and Asian (2%). The average age at preschool entry was approximately 55 months (M = 54.39, SD = 3.89). Children had a median of one sibling (IQR = 1–2), and on average, 12% had at least one immigrant parent, and 29% resided in rural counties. The integration of health and education records also allowed comparison of the matched cohort to the whole populations of children who were born in the state or who attended kindergarten to demonstrate representativeness of the final sample. In terms of individual risk factors, nearly half (49%) of the children were from households receiving public assistance (Special Supplemental Nutrition Program for Women, Infants, and Children [WIC] and Medicaid), 8% were born preterm or with low birthweight, 9% had mothers with low levels of education, and 36% were born to unmarried mothers. Additional risk factors included inadequate prenatal care (7%), maternal smoking during pregnancy (23%), and being born to a teen mother (7%). With respect to cumulative risk, 39% of children had no identified risks, 18% had one risk, another 19% had two risks, and 25% were exposed to three or more cumulative risks.

**Table 1:** Description of the Integrated Cohort

	**Matched all**	**VS only**	**DE only**	**Matched vs non-matched OR (SE)**	
	**Mean (SD)**	**Mean (SD)**	**Mean (SD)**	**Odds ratio**	**Sig.**
**Child characteristics**					
Male	51.0%			0.94	**
**Race/Ethnicity**					
White	78.0%		59.0%		
Black	5.0%		15.0%	0.26	***
Hispanic	10.0%		13.0%	0.61	***
Asian	2.0%		7.0%	0.22	***
Multiple	5.0%		5.0%	0.77	***
Age at preschool	54.39 (3.89)		54.63 (4.03)	0.98	***
Poor Attendance	7.5%		12.0%	0.72	***
Suspension	1.0%		1.3%	1.09	
**Family Characteristics**					
Parent immigration	12.0%	16.0%		0.77	***
Number of siblings	1 (1-2)	1 (1-2)		0.91	***
Rural	29.0%	27.0%	8.0%	1.06	*
**Individual risks**					
Poverty (WIC/Medicaid)	49.0%	43.0%		1.13	*
Preterm/LBW	8.0%	8.0%		0.92	ns
Low mother education	9.0%	11.0%		0.79	***
Unmarried mother	36.0%	30.0%		1.06	ns
Inadequate prenatal care	7.0%	11.0%		0.52	***
Teen mother	7.0%	6.0%		0.76	***
Smoking	23.0%	17.0%		1.16	**
**Cumulative risk^a^**					
0 Risks	39.0%	43.0%			
1 Risk	18.0%	19.0%		1.09	ns
2 Risks	19.0%	17.0%		1.21	*
3 or more Risks	25.0%	21.0%		1.38	*

Note. ^*^p < 0.10, ^**^p < 0.05, ^***^p < 0.001. Sibling count is reported as median (IQR) due to non-normal distribution. Cumulative risk index is based on a sum of: Poverty (WIC/Medicaid receipt), Preterm/Low birthweight, Low mother education, Unmarried mother, Inadequate prenatal care, Teen mother, and Smoking. DE = Department of Education; VS = Vital Statistics.

### Unduplicated Number of Children with at Least One Centre-based ECE

Figure 1 presents the distribution of Iowa children across distinct ECE experiences during the 2016–2017 academic year. ECE participation was constructed by integrating three statewide administrative data systems: (1) Child Care Assistance (CCA) payment records, which indicate receipt of subsidised child care; (2) Department of Education (DE) preschool enrollment records, which capture participation in DE-funded preschool programs offered through school districts; and (3) Teaching Strategies GOLD (GOLD) records, which capture enrollment in programs participating in Iowa’s statewide early childhood assessment system (including district-affiliated preK and many Head Start/community-based providers). The figure displays 19,964 unique individuals, which is 73% of the 27,219 children in the matched cohort, where the remaining 27% had no ECE during the year prior to kindergarten entry. This unduplicated count underscores a central finding: although children appeared across multiple administrative systems, nearly three-quarters of the matched cohort were captured as having some early care experience.

**Figure 1:**
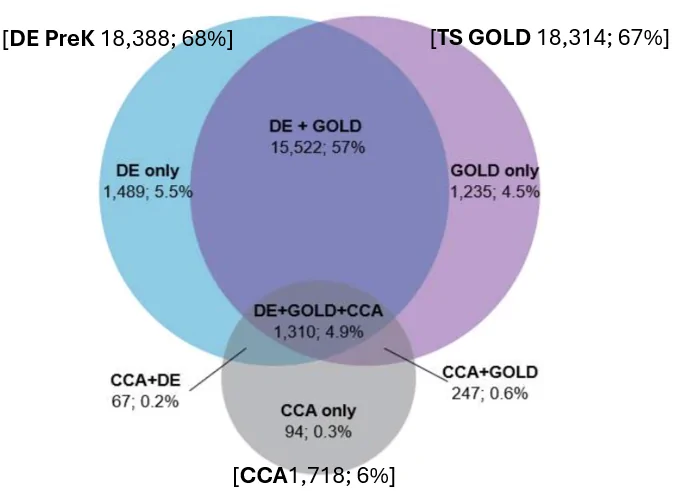
Preschool Experiences Among Children in the Birth-To-Five Cohort

*Note*: CCA = Child Care Assistance CCA; DE = Department of Education; GOLD = Teaching Strategies GOLD. Values in brackets indicate total N and percentage of the full study cohort for each dataset.

Exploration of overlap in preschool participation across these three data systems (DE, GOLD, and CCA) reveals important patterns. Overall, the majority of children were represented in either DE PreK or TS GOLD. Specifically, 18,388 children (68%) appeared in DE PreK, while 18,314 children (67%) were recorded in TS GOLD. A substantial proportion of children (57%) were captured in both systems (DE + GOLD; n = 15,522), indicating considerable overlap in enrollment reporting. Smaller groups were represented exclusively in one system: 5.5% in DE only (1,489 children), and 4.5% in GOLD only (1,235 children). Notably, the “GOLD only” group likely represents children who were not captured in DE or CCA but instead were enrolled in other preschool settings, such as Head Start or private preschool programs.

In comparison, fewer children were identified through CCA. A total of 1,718 children (6%) appeared in CCA records, though only 94 (0.3%) were uniquely identified in this system. Most CCA participants were also represented in DE or GOLD, with 1,310 children (4.9%) in all three systems (DE + GOLD + CCA), 247 (0.6%) in CCA + GOLD, and 67 (0.2%) in CCA + DE. These results highlight the value of integrated data for understanding the reach and overlap of ECE services. While DE and GOLD capture the majority of participants, CCA identifies a smaller but distinct group, particularly when linked with other systems.

Table 2 presents descriptive statistics of study variables by ECE type. DE + GOLD (the largest subset of the overall sample) was predominantly White (82%) with low poverty (44%) and lower risk exposure (20% with ≥ 3 risks). In contrast, CCA groups included higher proportions of Black and Hispanic children (22–24% Black; ∼20% Hispanic), nearly universal poverty (93–97%), and elevated risk exposure (47% with ≥3 risks). The GOLD only and DE only groups demonstrated intermediate risk profiles, while multi-program groups generally fell between the DE + GOLD and CCA only groups.

**Table 2:** Descriptive statistics of study variables by early care experience

	**DE+GOLD**	**DE only**	**GOLD only**	**DE+GOLD+CCA**	**CCA+DE**	**CCA+GOLD**	**CCA only**
**Child Characteristics**							
Male	51.0%	51.0%	52.0%	54.0%	60.0%	52.0%	49.0%
**Race/Ethnicity**							
White	82.0%	73.0%	67.0%	62.0%	58.0%	52.0%	36.0%
Black	3.0%	5.0%	11.0%	15.0%	16.0%	24.0%	22.0%
Hispanic	9.0%	16.0%	14.0%	11.0%	13.0%	9.0%	20.0%
Asian	2.0%	2.0%	2.0%	1.0%	1.0%	0.00%	1.0%
Multiple	4.0%	4.0%	6.0%	11.0%	10.0%	14.0%	20.0%
Age at preschool	54.16 (3.59)	54.46 (3.76)	54.26 (3.85)	54.06 (3.60)	54.33 (3.78)	53.98 (3.77)	53.43 (3.79)
**Family characteristics**							
Parent immigration	11.0%	17.0%	16.0%	8.0%	3.0%	5.0%	12.0%
Number of siblings	1.04 (1.09)	1.09 (1.17)	1.12 (1.26)	1.16 (1.25)	1.36 (1.37)	1.33 (1.65)	1.13 (1.34)
Rural	32.0%	38.0%	38.0%	31.0%	33.0%	37.0%	24.0%
**Individual risks**							
Poverty (WIC/Medicaid)	44.0%	55.0%	73.0%	91.0%	97.0%	96.0%	93.0%
Preterm/LBW	8.0%	8.0%	9.0%	8.0%	6.0%	11.0%	12.0%
Low mother education	7.0%	13.0%	15.0%	11.0%	6.0%	8.0%	15.0%
Unmarried mother	31.0%	41.0%	54.0%	76.0%	81.0%	76.0%	83.0%
Inadequate prenatal care	6.0%	7.0%	10.0%	10.0%	9.0%	12.0%	17.0%
Teen mother	5.0%	8.0%	12.0%	15.0%	16.0%	19.0%	31.0%
Smoking	12.0%	24.0%	33.0%	40.0%	48.0%	45.0%	40.0%
**Cumulative risk^a^**							
0 Risks	42.0%	32.0%	20.0%	3.0%	1.0%	2.0%	1.0%
1 Risk	20.0%	19.0%	14.0%	12.0%	9.0%	11.0%	7.0%
2 Risks	18.0%	21.0%	24.0%	32.0%	34.0%	26.0%	24.0%
3 or more Risks	20.0%	28.0%	43.0%	52.0%	55.0%	61.0%	67.0%

*Note*: ^a^ Cumulative risk index created using Preterm/Low birthweight, Teen mother, Low mother education, Unmarried mother, Inadequate prenatal care, WIC/Medicaid receipt, and Smoking. CCA = Child Care Assistance CCA; DE = Department of Education; GOLD = Teaching Strategies GOLD.

### Child and Family Characteristics and ECE Participation

#### Individual Risks

As shown in Table 3, individual child and family characteristics was associated participation in ECE programs. Poverty demonstrated the strongest association with participation patterns across systems. Compared to children not receiving Medicaid or WIC, children in families receiving Medicaid or WIC had 7.20 times greater odds of participating in CCA (p < 0.001) and modestly higher odds of appearing in DE (OR = 1.15, p < 0.001), GOLD (OR = 1.33, p < 0.001), and any ECE program (OR = 1.43, p < 0.001). Other health-related characteristics showed smaller associations. For example, compared to children not born preterm or low birthweight, children born preterm or low birthweight had modestly higher odds of GOLD participation (OR = 1.13, p < 0.05) and participation in any ECE program (OR = 1.17, p < 0.01).

**Table 3:** Odds Ratios of Child Characteristics and Individual Risks Related to ECE Participation in the Year Before Kindergarten

	**Child care**		**Department of**		**Teaching**		**Any ECE**	
	**assistance**		**education**		**strategies**		**(CCA, DE,**	
	**(CCA)**		**(DE)**		**GOLD**		**or GOLD)**	
	**Odds ratio**	**Sig.**	**Odds ratio**	**Sig.**	**Odds ratio**	**Sig.**	**Odds ratio**	**Sig.**
**Child Characteristics^a^**								
Male	1.10	ns	1.00	ns	1.00	ns	1.00	ns
**Race/Ethnicity**								
Black	2.63	***	0.58	***	0.77	***	0.82	**
Hispanic	1.18	ns	0.85	**	0.78	***	0.87	*
Asian	0.63	ns	1.00	ns	1.06	ns	1.05	ns
Multiple	1.79	***	0.71	***	0.79	***	0.78	***
Age at preschool	0.99	ns	0.96	***	0.95	***	0.95	***
**Family characteristics**								
Parent immigration	0.53	***	0.95	ns	0.89	*	0.91	ns
Number of siblings	1.12	***	1.01	ns	1.02	ns	1.03	*
Rural	0.98	ns	1.72	***	1.67	***	2.08	***
**Individual Risks^b^**								
Poverty (Medicaid/WIC)	7.20	***	1.15	***	1.33	***	1.43	***
Preterm or low birth weight	1.00	ns	1.08	ns	1.13	*	1.17	**
Low maternal education	0.68	***	0.78	***	0.76	***	0.76	***
Unmarried mother	2.17	***	0.94	ns	0.96	ns	1.00	ns
Inadequate prenatal care	0.92	ns	0.84	**	0.87	**	0.85	**
Teen mother	1.34	***	0.77	***	0.85	**	0.89	ns
Prenatal smoking	1.23	***	0.89	**	0.95	ns	0.96	ns
Intercept	0.01	***	22.23	***	22.21	***	32.31	***
Pseudo R-Squared	0.17		0.02		0.02		0.03	

*Note*: ^+^p<.10; ^*^p<.05; ^**^p<.01; ^***^p<.001. ^a^ Reference category is white, female. ^b^ Reference category is a child with zero of the listed risks.

Children of teen mothers also exhibited different participation profiles across systems. Compared to children of non-teen mothers, children of teen mothers had higher odds of CCA participation (OR = 1.34, p < 0.001) but lower odds of enrollment in DE (OR = 0.77, p < 0.001) and GOLD (OR = 0.85, p < 0.01). Similarly, compared to children whose mothers did not smoke during pregnancy, prenatal smoking was associated with higher odds of CCA participation (OR = 1.23, p < 0.001) and lower odds of DE enrollment (OR = 0.89, p < 0.01). By contrast, compared to children whose mothers had higher educational attainment, low maternal education was associated with lower participation across systems (ORs = 0.68–0.78, p < 0.001). Children of unmarried mothers had higher odds of appearing in CCA (OR = 2.17, p < 0.001) and lower odds of appearing in DE and GOLD compared to children of married mothers.

Demographic characteristics were also associated with ECE participation. Compared to White children, Black children had substantially higher odds of CCA participation (OR = 2.63, p < 0.001) but lower odds of enrollment in DE (OR = 0.58, p < 0.001) and GOLD (OR = 0.77, p < 0.001). Relative to White children, Hispanic and multi-racial children showed a similar pattern in direction (higher odds of CCA participation, though not statistically significant), while exhibiting significantly lower odds of DE and GOLD enrollment. Compared to children of non-immigrant parents, children of immigrant parents had significantly lower odds of appearing in CCA (OR = 0.53, p < 0.001) or GOLD (OR = 0.89, p < 0.05). Finally, compared to non-rural children, rural children had markedly higher odds of participating in DE (OR = 1.72, p < 0.001), GOLD (OR = 1.67, p < 0.001), and any ECE program (OR = 2.08, p < 0.001).

#### Cumulative Risks

As shown in Table 4, participation in ECE programs was associated with children’s exposure to cumulative birth risks in the year prior to kindergarten. Compared to children with no risks, children with one risk had 1.35 times greater odds of enrollment in any ECE program (p < 0.001), and children with two risks had 1.48 times greater odds (p < 0.001). Children with three or more risks also had higher odds of enrollment relative to those with no risks (OR = 1.23, p < 0.001), although the magnitude of this association was smaller than for children with one or two risks. Overall, the model explained a modest proportion of variance (Pseudo R^2^ = .03), suggesting that additional factors beyond cumulative risk are associated with ECE enrollment. Models using individual versus cumulative risk indicators demonstrated nearly identical explanatory power.

**Table 4:** Likelihood of Children Participating in Each Experience by Cumulative Risk

	Child Care		Department of		Teaching		Any ECE	
	Assistance (CCA)		Education (DE)		Strategies GOLD		(CCA, DE, or GOLD)	
**Child characteristics^a^**								
Male	1.10	ns	1.00	ns	1.00	ns	1.00	ns
**Race/Ethnicity**								
Black	2.94	ns	0.59	***	0.78	***	0.84	*
Hispanic	1.22	***	0.83	***	0.77	***	0.85	**
Asian	0.58	*	1.00	ns	1.05	ns	1.04	ns
Multiple	1.98	ns	0.72	***	0.80	***	0.79	***
Age at preschool	0.99	***	0.96	***	0.95	***	0.95	***
**Family characteristics**								
Parent immigration	0.40	***	0.91	*	0.85	**	0.86	**
Number of siblings	1.07	**	1.01	ns	1.02	ns	1.03	*
Rural	1.04	ns	1.71	***	1.67	***	2.09	***
**Cumulative risks^b^**								
1 Risk	8.73	***	1.23	***	1.25	***	1.35	***
2 Risks	21.56	***	1.18	***	1.31	***	1.48	***
3 or more Risks	27.99	***	0.86	***	1.09	*	1.23	***
Intercept	0.01	***	21.63	***	22.12	***	32.28	***
Pseudo R-Squared	0.16		0.02		0.02		0.03	

*Note*: ^+^p<.10; ^*^p<.05; ^**^p<.01; ^***^p<.001. ^a^ Reference category is white, female. ^b^ Reference category is a child with zero of the listed risks.

Associations also differed by ECE program type. For CCA, cumulative risk was strongly associated with participation: compared to children with no risks, children with one, two, and three or more risks had substantially higher odds of participation (ORs = 8.73, 21.56, and 27.99, respectively; all p < 0.001). For DE programs, children with one or two risks had modestly higher odds of participation relative to children with no risks (OR = 1.23 and 1.18, both p < 0.001), whereas children with three or more risks had lower odds of participation (OR = 0.86, p < 0.001). For TS GOLD, children with one or two risks had higher odds of enrollment compared to children with no risks (ORs = 1.25 and 1.31, both p < 0.001), while children with three or more risks had only slightly higher odds (OR = 1.09, p < 0.05).

Several demographic and family characteristics were also associated with participation patterns. Compared to White children, Black children had substantially higher odds of participating in CCA (OR = 2.94, p < 0.001) but lower odds of participation in DE (OR = 0.59, p < 0.001) and GOLD (OR =  0.78, p < 0.001). Hispanic and multi-racial children exhibited a similar pattern relative to White children: higher odds of CCA participation and lower odds of participation in DE and GOLD programs. Compared to children of non-immigrant parents, children of immigrant parents had lower odds of participation in DE, GOLD, and any ECE (ORs = 0.85–0.91), and substantially lower odds of participating in CCA (OR = 0.40, p < 0.001). Finally, compared to non-rural children, rural children had higher odds of enrollment across systems, particularly in DE (OR = 1.71, p < 0.001) and GOLD (OR = 1.67, p < 0.001).

### ECE Enrollment and Kindergarten Attendance and Behaviour

Findings from logistic regression analyses indicated that participation in any ECE during the year prior to kindergarten was associated with differences in attendance, but not suspension outcomes. Any ECE was defined as those appearing in at least one of the three statewide administrative sources (CCA, DE, and/or GOLD), with the reference group comprising children with no observed ECE participation in any of these systems (see Table 5). Specifically, children with any ECE participation had lower odds of poor attendance in kindergarten (OR = 0.82, p < 0.10), reflecting an approximately 18% reduction in the odds. Several covariates were also associated with kindergarten outcomes. Poverty at birth was associated with substantially higher odds of poor attendance (OR = 2.80, p < 0.001) and suspensions (OR = 2.86, p < 0.001). Boys also had markedly higher odds of suspension compared to girls (OR = 7.52, p < 0.001).

**Table 5:** Odds Ratios of Child Characteristics and Cumulative Risks Related to Kindergarten Indicators

	**Poor attendance**		**Suspensions**	
	**Odds ratio**	**Sig.**	**Odds ratio**	**Sig.**
**Child Characteristics^a^**				
Male	1.06	ns	7.52	***
**Race/Ethnicity**				
Black	2.18	***	3.82	***
Hispanic	1.74	***	0.84	ns
Asian	2.05	***	1.26	ns
Multiple	1.41	***	2.35	***
Age at preschool	1.00	ns	1.00	ns
**Family characteristics**				
Parent immigration	0.89	ns	0.52	*
Number of siblings	1.03	ns	1.09	ns
Rural	1.03	ns	0.87	ns
**Individual Risks^b^**				
Poverty (Medicaid/WIC)	2.80	***	2.86	***
Preterm or low birth weight	1.16	ns	0.70	ns
Low maternal education	1.39	***	0.95	ns
Unmarried mother	1.29	***	1.14	ns
Inadequate prenatal care	1.22	*	1.18	ns
Teen mother	1.80	***	1.39	ns
Prenatal smoking	1.53	***	1.61	***
Any public preschool	0.82	***	1.16	ns
Intercept	0.03	***	0.00	***
Pseudo R-Squared	0.09		0.13	

*Note*: ^*^p < 0.10, ^**^p < 0.05, ^***^p < 0.001. ^a^ Reference category is white, female. ^b^ Reference category is a child without the noted risk.

Similar patterns were observed in the cumulative risk models (see Table 6). Children who participated in any ECE continued to show lower odds of poor attendance in kindergarten (OR = 0.82, p < 0.001), though the magnitude of the association remained modest.

**Table 6:** Odds Ratios of Child Characteristics and Individual Risks Related to Kindergarten Indicators

	**Poor attendance**		**Suspensions**	
	**Odds ratio**	**Sig.**	**Odds ratio**	**Sig.**
**Child Characteristics^a^**				
Male	1.05	ns	7.49	***
**Race/Ethnicity**				
Black	2.23	***	3.71	***
Hispanic	1.78	***	0.80	ns
Asian	2.04	***	1.18	ns
Multiple	1.44	***	2.38	***
Age at preschool	1.00	ns	1.00	ns
**Family characteristics**				
Parent immigration	0.85	*	0.48	**
Number of siblings	1.03	ns	1.09	*
Rural	1.08	ns	0.93	ns
**Cumulative risks^b^**				
1 Risk	2.63	***	1.84	*
2 Risks	4.09	***	3.63	***
3 or more Risks	6.89	***	4.72	***
Any public preschool	0.82	***	1.17	ns
Intercept	0.02	***	0.00	***
Pseudo R-Squared	0.09		0.13	

*Note*: ^*^p < 0.10, ^**^p < 0.05, ^***^p < 0.001. ^a^ Reference category is white, female. ^b^ Cumulative risk index created using Poverty (WIC/Medicaid receipt), Preterm/Low birthweight, Low mother education, Unmarried mother, Inadequate prenatal care, Teen mother, and Smoking.

As a sensitivity analysis, we re-estimated the models using multilevel logistic regression, with school districts (n = 333) specified as the level-2 clustering variable. The unconditional models yielded intraclass correlation coefficients (ICCs) of 0.08 for poor attendance and 0.29 for suspension. Results for both outcomes were largely unchanged and are presented in the Appendix (Tables A1 and A2).

## Discussion

This study leveraged an integrated data system including health and education records to document, for the first time in the state of Iowa, unduplicated counts of children in ECE programming. By drawing on a comprehensive set of longitudinal, linked administrative records, we provide new evidence on how early-life risks, family characteristics, and participation in different ECE programs are related to children’s suspension and attendance outcomes in their first year of formal schooling. This approach offers a unique vantage point, as few states currently have the infrastructure to link child-level data across multiple programs and service systems at this scale.

Three central findings emerged that contribute to the literature about ECE participation and showcase the value of the integrated data system approach. First, most children in the study sample (73%) participated in at least one centre-based ECE program prior to kindergarten entry. This participation pattern aligns with prior descriptions of centre-based enrollment among children preparing to enter kindergarten [43] and extends this research by providing a much more nuanced look at the varied types and patterns of enrollment. Namely, drawing on multiple sources of data from public education, health, and child care, we were able to document unique patterns of participation that reveal how children engage with ECE across public, subsidised, and private systems.

We find that most children were captured in both the state’s voluntary preschool program and the child assessment system, but clearer patterns emerge when we conceptualise children in broader groups rather than by acronyms alone. The largest group—which represents “all-care” children (DE + GOLD + CCA)–appears across publicly funded preschool, the child assessment system, and child care subsidies. These children likely come from families piecing together full-day arrangements through multiple public supports. This group exhibits the highest levels of socioeconomic disadvantage: 91% lived in poverty, compared with 44–55% of children in preschool-only groups, and they experienced elevated rates of unmarried mothers, teen mothers, maternal smoking, and exposure to multiple cumulative risks. These patterns suggest that participation across multiple systems is concentrated among families facing significant structural disadvantage.

A second group–children enrolled in public preschool and observed in the child assessment system but not receiving subsidies (“public PreK only”; DE + GOLD)–appears comparatively more advantaged. These children had lower poverty rates, higher parental education, and fewer cumulative risks, suggesting that participation in public prekindergarten without subsidy reliance may reflect somewhat greater family resources.

At the margins, 5.5% of children were enrolled in public preschool-only (DE only), while 4.5% appeared only in the child assessment system, “unsubsidised” or private-care children (GOLD only). The unsubsidised/private-care group (GOLD only) exhibited a higher proportion of exposure to two or more risks relative to those in the public preschool-only (DE only) and public PreK plus assessment (DE + GOLD) groups. This is noteworthy because these children likely include those enrolled in private preschool settings who are not participating in the state’s voluntary preschool program or receiving child care subsidies. The concentration of risk within this group suggests that disadvantage is not confined to publicly funded systems and that some vulnerable children may be served outside formal public early education structures. This pattern may reflect family preferences for private, full-day programs, or structural constraints such as limited availability of full-day public options that push working families toward private-care.

Finally, a small but important subgroup (0.3%) appeared only in child care subsidies (“subsidy-only”; CCA only). Although numerically small, this group raises critical questions about why subsidy-eligible children are not accessing public preschool–whether due to program availability, transportation barriers, scheduling constraints, or parental choice. Taken together, these findings demonstrate how an integrated data system allows us to move beyond program silos and instead identify meaningful constellations of care participation and risk exposure–revealing both concentrations of disadvantage and potential gaps in service alignment across health, education, and subsidy systems.

When participation was examined across all early care types, additional disparities emerged. Poverty emerged as the strongest and most consistent predictor of ECE category membership, underscoring the extent to which economic disadvantage is a significant factor related to family opportunities for children’s early care experiences. In addition, children from racially minoritised backgrounds, those born to mothers with lower educational attainment, and those whose mothers received inadequate prenatal care were significantly less likely to participate in ECE of any kind. Although the estimated variance explained in the “any care” model (Any ECE) are modest in magnitude–approximately a 3-percentage-point change–they warrant consideration in context. In large, population-level administrative datasets, even small percentage differences can translate into meaningful shifts in the number of children affected [44]. From a policy perspective, effects of this size may represent substantial changes when scaled across cohorts or geographic areas. At the same time, we interpret these findings cautiously and acknowledge that, while statistically significant, the practical significance at the individual-level is modest.

Further, these inequities observed in this model are especially concerning given that these same groups of children often face elevated developmental risks and may stand to benefit the most from the supports provided in early learning environments [39, 45] Importantly, in this state the public PreK options are offered as a half-day program 4 days per week. As findings suggest those who participate may be more advantaged, it raises questions about access and whether such a program is fully meeting the needs of children who already face disadvantages related to socioeconomic factors. Additionally, findings from this study highlight that children living in rural areas were more likely to participate in ECE compared to their urban counterparts. This could also reflect a relative shortage of ECE options in urban areas compared to rural areas, a finding consistent with existing literature [46].

Beyond these differences, findings also suggest that cumulative early-life risk factors were associated with patterns of early care participation, regardless of the type of risk. These findings are consistent with prior research showing that early care and education participation reflects both family need and structural access. Children with one or two identified risks were more likely to participate in early care programs, which may reflect eligibility pathways for publicly funded programs such as Head Start, state prekindergarten, and Child Care Assistance [47]. Prior work suggests that among low-income families, factors that increase the need for care can also increase the likelihood of preschool enrollment [48] and that child care subsidy policies shape families’ use of centre-based care [49].

At the same time, our finding that participation was relatively lower among children experiencing three or more risks suggests that accumulated disadvantage may create barriers to access or sustained participation. This interpretation aligns with literature documenting structural barriers to equitable ECE access, including affordability, limited supply, administrative complexity, transportation challenges, work schedule instability, and linguistic or immigration-related barriers [50]. Thus, the observed pattern may reflect a curvilinear relationship: moderate levels of risk may increase contact with targeted early care systems, whereas more concentrated risk may make program access and participation more difficult. In this way, the findings suggest that early care systems may successfully reach many children experiencing disadvantage, while still leaving some of the most multiply burdened families at risk of being underserved. This is consistent with theoretical and empirical literature suggesting higher cumulative risk compounds disadvantages [25, 51], creating barriers including transportation challenges, competing family stressors, or limited local program availability that may reduce the likelihood of ECE participation.

A third contribution is the study’s use of integrated data systems to show that participation in public ECE programs was significantly associated with more favourable kindergarten outcomes, underscoring the potential of linked administrative data to illuminate early pathways into school success. Specifically, children who attended ECE programs demonstrated better attendance during their first year of school. The association between public ECE participation and lower odds of poor kindergarten attendance is consistent with prior research showing that early attendance patterns are established before or during the transition to formal schooling. Studies of preschool attendance have found that levels of attendance in prekindergarten is associated with continued attendance in kindergarten and the early elementary grades [52]. This finding points to the value of ECE as a potentially important lever for facilitating consistent school attendance from the start of formal education. Importantly, this associations remained robust even after accounting for individual early health risks and family demographics, suggesting that ECE participation exerts a protective influence that supports smoother transitions into formal schooling [39, 53]. Importantly, relationships were not found between ECE participation and kindergarten suspensions. This finding adds nuance to prior work that has raised concerns about links between formal ECE participation and later behaviour problems [54]. In our study, children with formal ECE experiences did not exhibit behaviour concerns of sufficient magnitude to result in kindergarten suspension, suggesting that ECE participation–particularly in contexts that may reflect supportive developmental practices–does not necessarily translate into severe behavioural challenges at school entry.

These findings contribute to the growing body of evidence suggesting early childhood education not only prepares children academically but also fosters the behavioural and social foundations critical for sustained attendance in school [55]. Improved attendance in kindergarten among children who attended ECE suggests that early participation helps establish consistent routines of showing up, which research identifies as a key marker of engagement. Children from socioeconomically disadvantaged backgrounds, in particular, show stronger literacy gains when they maintain good attendance, suggesting that consistent school exposure may help narrow early skill gaps [56]. At the same time, chronic absenteeism in kindergarten has been linked not only to lower achievement but also to long-term declines in executive functioning, including working memory and cognitive flexibility–skills essential for sustained learning [57]. More recent work further underscores the importance of preschool participation in supporting attendance beyond the early grades: in Tulsa, public preschool attendance was associated with meaningful reductions in third-grade absenteeism for dual language learners in the post-COVID-19 era, providing a potential strategy to counter rising absenteeism rates [58].

### Limitations and Recommendations for Future Research

Despite its contributions, this study is not without limitations. First, the analyses draw on data from a single state. Iowa provides a compelling case study because of its strong data infrastructure and relatively well-developed early childhood policy landscape, yet the findings may not readily generalise to other contexts where demographic characteristics, political priorities, or education systems differ. In the United States, for example, there is considerable variation in how individual states fund and deliver public preschool, regulate and assess child care quality, and structure eligibility for other early education programs such as those designed for low-income or disadvantaged families. Replicating the integrated data system approach in states with more racially and ethnically diverse populations, different levels of economic inequality, or distinct child care policy frameworks would help test the robustness and generalizability of the findings. Cross-state and international comparisons could also illuminate how policy levers at federal, state, and local levels interact with family and community characteristics to influence access and outcomes.

Second, the study does not distinguish some specific program types such as Head Start from other ECE options. This is an important omission given that Head Start in the United States serves a disproportionately high percentage of Black children and plays a unique role in supporting families facing economic hardship [12]. Identifying Head Start participants separately would allow researchers to examine whether program effects differ across demographic groups and whether the program’s targeted supports translate into distinct outcomes relative to state-funded preschool or private-sector care. More broadly, the analyses do not incorporate variation across delivery models (e.g., full-day versus part-day programs, school-based versus community-based centres, public versus private providers) or program quality indicators such as participation in quality rating and improvement systems. Without this detail, the study cannot fully capture the heterogeneity of children’s early education experiences or assess whether certain models or quality levels are more effective in promoting school readiness. Future work that integrates these programmatic dimensions with the rich data contained in public health and social service records would provide a more nuanced understanding of how early education systems serve diverse populations.

Third, the study does not account for community-level health and education contexts that may shape both access to early education and children’s subsequent outcomes. In addition to neighbourhood poverty and employment rates, factors such as access to paediatric health care providers, immunisation coverage, participation in SNAP and WIC, food access and nutrition environments, housing stability, and transportation infrastructure may meaningfully influence children’s early development and school readiness. These broader community health indicators likely interact with early education access to structure opportunities for young children and should be incorporated into future cross-sector analyses [57, 59, 60]. Excluding these contextual health and service environments risks overlooking key mechanisms that may either exacerbate or buffer the associations observed at the family and child levels. For example, even high-quality preschool programs may yield diminished benefits when children lack consistent access to primary health care, fall behind on immunisations, experience food insecurity, or live in communities with limited SNAP/WIC uptake or poor transportation infrastructure. Conversely, strong local health systems and nutrition supports may amplify the benefits of early education. Incorporating these cross-sector ecological dimensions would allow future research to more fully situate preschool participation within the broader health and education systems that shape children’s developmental trajectories.

Looking ahead, several extensions of this work would strengthen the evidence base for educational policy and practice. Using this study as a model for the integration of health and education data, replicating and expanding analyses across multiple sites would allow for cross-contextual comparisons that highlight how variations in policy, program design, and demographic composition shape access and outcomes. As integrated data systems continue to evolve internationally, cross-cultural studies integrating vital statistics health records with ECE enrollment could identify characteristics of families and communities that best facilitate access to ECE. Disaggregating program types and delivery models using the rich data contained within administrative records would help identify which approaches are most effective for which populations, providing guidance for tailoring investments. Additionally, longitudinal follow-up beyond kindergarten entry is essential to assess the persistent and long-term impact of early education, particularly for children from high-risk or underserved populations. By addressing these gaps, future research can provide policymakers with more precise, actionable evidence to promote equitable access to high-quality early learning opportunities.

### Implications for Practice and Policy

These findings carry several actionable implications for policy and practice to advance early childhood education priorities. First, federal and state government leadership could prioritise the development and maintenance of comprehensive, integrated data systems capable of generating unduplicated child-level participation counts across the myriad of ECE programs. Given the fragmented nature of ECE, that is an international phenomenon not unique to the United States, such systems are critical for understanding who is being served across programs, where gaps in access or quality exist, and how resources can be better allocated to meet the needs of all families with young children. In the absence of integrated data infrastructures, policymakers and administrators will continue to face major challenges in identifying overlapping enrollment, assessing unmet need, and targeting supports to children and families who might otherwise fall through the cracks [61]. Moreover, integrated data systems facilitate the quicker and accurate use of data by ensuring that information is systemically collected, organised, and readily accessible. This allows for real-time, evidence-based decision-making and program evaluation that is not available from one-off research projects or costly and time-consuming direct data collection efforts that are currently more typical in early education research [2].

In addition to the value of integrated data, the findings have some direct implications for programming in early childhood. Specifically, they underscore the need for greater outreach and tailored supports for families experiencing the highest levels of cumulative risk that can be identified using public health records at the time of birth. These families are often those most in need of early education opportunities, yet they face the greatest barriers to accessing them, whether due to logistical challenges, eligibility complexity, or lack of awareness [62–64]. Expanding coordinated enrollment systems that are situated at birthing hospitals or other places young families already engage for services could help connect families with services earlier. Streamlining eligibility requirements across programs and implementing culturally responsive communication strategies are other concrete ways that could reduce barriers for families who are currently less likely to engage in ECE. Moreover, policies that provide wraparound supports such as transportation assistance, flexible scheduling, and family engagement programs could further enhance accessibility and participation [65]. Finally, ongoing efforts to strengthen both quality and coordination across early childhood programs remain vital. In the United States, there continues to be fragmented guidelines and accountability frameworks guiding different ECE programs. Aligning Head Start, Child Care Assistance, and state-funded PreK, for example, under shared quality frameworks can reduce fragmentation and ensure that all children, regardless of the program they attend, receive a baseline of high-quality care and education.

Taken together, these implications highlight the importance of building systems that are not only data-informed but also equity-focused and family-centred. As public investments in early childhood education expand, policies must move beyond simply increasing access to ensure that access is meaningful, equitable, and tied to high-quality experiences. Only then can the promise of universal, high-quality PreK be fully realised and translated into lasting developmental benefits for children, particularly those from underserved and high-risk populations.

## Conclusion

This study highlights the power of integrating education and health administrative data to generate actionable intelligence about children’s early experiences and school readiness. By leveraging Iowa’s integrated data system, we identified who participates in preschool, how early program access connects to early disciplinary and attendance patterns, and where gaps remain. More broadly, our findings underscore the value of building and sustaining cross-sector data systems in partnership with policymakers–systems that not only advance research, but directly inform targeted, evidence-based decisions that improve outcomes for children and families.

These findings also demonstrate how actionable intelligence can inform policy beyond a single context and motivate other countries, states and jurisdictions to develop similar administrative data capacity. Leveraging integrated administrative education and health data systems provides a scalable framework for broader implementation and cross-jurisdictional learning. Illustrations of this can be seen in other states and municipalities. For example, Wisconsin-based IDS research demonstrating that policy changes reducing interest rates on child support arrears were associated with higher payment rates informed legislative efforts in Texas to lower arrears interest rates [15]. Similarly, findings from Philadelphia’s IDS were used to evaluate the effects of the passage of a soda tax in neighbourhoods across Philadelphia [66]. Together, these examples underscore the broader point motivating our study: integrated administrative data systems can serve as powerful policy tools, translating rigorous empirical evidence into actionable reforms that directly shape the wellbeing of children and families.

We should continue investing in integrated data systems at multiple levels of government, as this study has demonstrated the value of the information they can provide to help reduce access barriers especially for the most vulnerable families. Importantly, an equity-driven approach is essential to ensure that children with the greatest needs are prioritised rather than left behind.

## Supplementary Files

Supplementary Appendices

## Data Availability

The data used in this study were obtained through Iowa’s Integrated Data System for Decision-Making (I2D2) and include linked administrative records provided by state agencies. Because the data contain sensitive individual-level information and are subject to legal agreements, privacy protections, and data-governance requirements, they are not publicly available and cannot be shared by the authors. Access may be considered through I2D2’s established project review and governance process, subject to agency approval, applicable data-use agreements, and authorization for research use.
