# Supplementary material for: Who and How Many? Using Integrated Health and Education Data to Study Early Care Experiences of Children in the US
Source: Int J Popul Data Sci. 2026 Aug 17;11(2):3366. doi: 10.23889/ijpds.v11i2.3366 (PMC13678713; doi:10.23889/ijpds.v11i2.3366)
Supplement: Supplementary file 1 [file ijpds-11-3366-s001.pdf]

Appendix

Table A1: Odds ratios of child characteristics and individual risks related to kindergarten indicators using multilevel modelling

	Poor attendance[d]		Suspensions[e]	
	**Odds ratio**	**Sig.**	**Odds ratio**	**Sig.**
**Fixed effects**				
**Child characteristics[a]**				
Male	1.05	ns	7.52	***
**Race/Ethnicity**				
Black	2.21	***	2.64	***
Hispanic	1.74	***	0.65	ns
Asian	2.17	***	0.94	ns
Multiple	1.42	***	1.88	***
Age at preschool	1.00	ns	1.00	ns
**Family characteristics**				
Parent immigration	1.14	ns	0.57	*
Number of siblings	1.03	ns	1.09	ns
Rural	0.90	ns	0.81	ns
**Individual risks[b]**				
Poverty (Medicaid/WIC)	2.81	***	2.38	***
Preterm or low birth weight	1.14	ns	0.67	ns
Low maternal education	1.40	***	0.89	ns
Unmarried mother	1.30	***	1.07	ns
Inadequate prenatal care	1.21	*	1.24	ns
Teen mother	1.82	***	1.34	ns
Prenatal smoking	1.54	***	1.51	***
Any public preschool	0.80	***	1.18	ns
Intercept	0.03	***	0.00	***
**Level 2 random effects**				
Var (_cons)[c]	0.12		0.96	

Note: $^{*}p < 0.10$, $^{**}p < 0.05$, $^{***}p < 0.001$. [a]Reference category is white, female. [b]Reference category is a child without the noted risk. [c]Cluster variable is school districts.[d]Intra Class Correlation (ICC) =0.08. [e]Intra Class Correlation (ICC) =0.29.

Table A2: Odds ratios of child characteristics and cumulative risks related to kindergarten indicators using multilevel modelling

	Poor attendance[d]		Suspensions[e]	
	**Odds ratio**	**Sig.**	**Odds ratio**	**Sig.**
**Fixed effects**				
**Child characteristics[a]**				
Male	1.05	ns	7.50	***
**Race/Ethnicity**				
Black	2.26	***	2.55	***
Hispanic	1.77	***	0.61	ns
Asian	2.14	***	0.89	ns
Multiple	1.45	***	1.88	***
Age at preschool	1.00	ns	1.00	ns
**Family characteristics**				
Parent immigration	0.93	ns	0.53	*
Number of siblings	1.03	ns	1.08	ns
Rural	0.95	ns	0.93	ns
**Cumulative risks[b]**				
1 Risk	2.64	***	1.61	ns
2 Risks	4.13	***	2.88	***
3 or more Risks	6.97	***	3.41	***
Any public preschool	0.80	***	1.17	ns
Intercept	0.03	***	0.00	***
**Level 2 random effects**				
Var (_cons) [c]	0.13		0.98	

Note: *$p < 0.10$, **$p < 0.05$, ***$p < 0.001$. [a] Reference category is white, female. [b] Cumulative risk index created using Poverty (WIC/Medicaid receipt), Preterm/Low birthweight, Low mother education, Unmarried mother, Inadequate prenatal care, Teen mother, and Smoking. [c] Cluster variable is school districts.[d] Intra Class Correlation (ICC) = 0.08. [e] Intra Class Correlation (ICC) = 0.29.
